# High-Throughput Sequencing of 16S rRNA Gene Amplicons: Effects of Extraction Procedure, Primer Length and Annealing Temperature

**DOI:** 10.1371/journal.pone.0038094

**Published:** 2012-05-29

**Authors:** Martin J. Sergeant, Chrystala Constantinidou, Tristan Cogan, Charles W. Penn, Mark J. Pallen

**Affiliations:** 1 Centre for Systems Biology, School of Biosciences, University of Birmingham, Birmingham, United Kingdom; 2 Mucosal Microbiology, School of Veterinary Sciences, University of Bristol, Bristol, United Kingdom; University of Vienna, Austria

## Abstract

The analysis of 16S-rDNA sequences to assess the bacterial community composition of a sample is a widely used technique that has increased with the advent of high throughput sequencing. Although considerable effort has been devoted to identifying the most informative region of the 16S gene and the optimal informatics procedures to process the data, little attention has been paid to the PCR step, in particular annealing temperature and primer length. To address this, amplicons derived from 16S-rDNA were generated from chicken caecal content DNA using different annealing temperatures, primers and different DNA extraction procedures. The amplicons were pyrosequenced to determine the optimal protocols for capture of maximum bacterial diversity from a chicken caecal sample. Even at very low annealing temperatures there was little effect on the community structure, although the abundance of some OTUs such as *Bifidobacterium* increased. Using shorter primers did not reveal any novel OTUs but did change the community profile obtained. Mechanical disruption of the sample by bead beating had a significant effect on the results obtained, as did repeated freezing and thawing. In conclusion, existing primers and standard annealing temperatures captured as much diversity as lower annealing temperatures and shorter primers.

## Introduction

Existing culture techniques are not routinely capable of capturing the total microbial diversity of complex microbial communities found in the intestinal tract of warm blooded animals. Hence culture independent, molecular techniques which analyse the DNA directly are often employed. In most cases, the 16S rRNA gene is amplified from total extracted DNA using ‘universal’ primers to target the conserved regions of the gene, and the resulting PCR products are sequenced to identify the bacterial species present. In pioneering work this was done by first genetically cloning individual molecules and sequencing these individually by conventional Sanger sequencing [1]. With the advent of high throughput sequencing, this approach to defining diversity in complex microbial populations has come into its own, since millions of sequences can now be obtained more cheaply and quickly. Pyrosequencing (Roche 454) in particular is attractive for 16S rRNA gene analysis because of the relatively long sequence reads obtained (500 bp) compared to other high throughput sequencing technologies. However there are caveats with 16S rRNA gene analysis: sample handling, DNA extraction [2], [3], PCR amplification [4], sequencing accuracy and data analysis [5] can all lead to distortion of the final result such that it does not reflect the true composition of the sample being analysed.

A critical step is the PCR, which employs so called ‘universal primers’ that anneal to conserved regions in the 16S rRNA gene and amplify as many 16S rRNA genes from different organisms as possible. Imperfect universal primers can preferentially amplify sequences containing an identical target region and even fail to amplify at all sequences which differ in the target region. An evaluation of the commonly used forward primer 27f has been carried out [6] and it was found that important groups such as the *Bifidobacteriales* contained mismatches in the primer target region. By incorporating degenerate bases and using a mix of primers the bias against the 16S rRNA gene of *Gardnerella*, a member of the *Bifidobacteriales* was reduced. However such primer mixes, presumably due to dilution of the main primer, lead to decreased amplification of the *Lactobacillus* 16S rRNA gene, for which the primers are an exact match. Apart from degenerate bases and using a mix of primers, another way to improve the binding of primers to imperfect target sequences is to lower the temperature of the annealing step of the PCR. Although the region of the 16S rRNA gene amplified [7] and the amplicon length [8], as well as the polymerase, cycle number and template dilution [4] have all been investigated using pyrosequencing, the effect of annealing temperature has been largely ignored. This is despite the fact that studies have shown that annealing temperature has a critical effect on the PCR, especially when there are mismatches between the primer and the target region on the template [9], [10]. Whether lowering the annealing temperature will increase or even result in new novel reads from bacterial species whose target regions differ from those of the universal primers is unknown.

Another potential improvement in ‘universal’ primer design is to reduce their length, thus there is less chance of a mismatch occurring with the target sequence, although there is a greater risk of amplifying not-target sequences. This was successfully achieved using ‘miniprimers’ of only 10 bp in length [11]. This approach has not been applied using pyrosequencing, nor to analysis of the intestinal microbiota. Again, next generation sequencing would nicely complement the use of miniprimers because with a larger number of reads, more spurious sequences can be tolerated. By using mini-primers that are designed to match the 5′ region of existing primers, the amplification of 16S rRNA genes that contain differences at the 3′ region of universal binding sites should be possible.

The processing of the sample is an important factor in analysis of its 16S rDNA gene content. Efficient lysis of all bacteria present is vital in order to obtain DNA from a true representation of organisms present. Often, samples cannot be processed immediately after collection on site and are flash frozen. Subsequently samples are homogenized before extraction and if the sample will be used in the future it is refrozen. Although the effect of different extraction procedures on 16S rRNA gene analysis has been evaluated using fingerprinting methods [2] and a phylogenetic microarray [3], it has not been investigated using pyrosequencing and neither has the effect of re-freezing the sample.

The chicken is the most important food production animal and the most abundant and widely distributed bird in the world. The microbiota are thought to play an essential role in the chicken and can effect growth performance and increase protection against pathogens [12]. Indeed, there are many techniques which aim to modify the microbiota in order to improve the growth performance of the chicken. These include the use of probiotics [13], prebiotics [14] and until recently growth promoting antibiotics [15]. However, precisely how the microbiota is altered by these methods, how this improves growth performance and which individual bacterial species are involved remains poorly understood. Recently molecular studies on the chicken microbiota have revealed candidate bacterial strains which improve growth performance [15] and which aid in exclusion of *Campylobacter* [16]. These studies have employed DGGE and array based methods, thus the use of more discriminative next generation sequencing should reveal more insight into the role of the microbiota in chicken health. However, the technique will only be successful if it can accurately reflect the true microbial composition of the chicken GIT. To this end, we studied the effect of changing primer length, annealing temperature and extraction conditions on the pyrosequencing of 16S rRNA gene sequences from chicken caecal samples.

## Results

### Sequence yields

The amplicons for all experiments (summarized in Table S1) were sequenced on two quarters of a 454 plate and yielded 329,326 sequences. However, about 2/3 of these reads were removed as they were below 400 flows after trimming, to leave 105,524 reads for further analysis. Table 1 shows the number of reads for each experimental condition and the percentage of chimeras and non-16S rRNA gene sequences detected. Supplemental tables S2, S3 and S4 show the number of reads comprising all OTUs in each sample and their assignment using the RDP classifier. For comparison, the closest BLAST match from the SILVA and NCBI nr databases along with percent identity are also shown.

**Table 1:** Summary of the reads obtained under different experimental conditions.

Conditions	Total Reads	Number after filtering	% non 16S reads	% chimeras	Total OTUs	Simpsons Index^a^
**Annealing Temperature**						
55°C	34623	13034	0	1.13	340	0.88
50°C	21487	7681	0	0.76	272	0.86
45°C	42252	13754	0.36	1.80	330	0.89
40°C	26768	9340	0.32	1.44	318	0.88
35°C	18919	6579	0.05	0.79	293	0.89
30°C	13857	6339	0.07	0.55	233	0.89
**Primers used**						
F14/R19	17229	4866	0.17	0.66	239	0.89
F20/R10	19348	6076	3.30	0.41	261	0.90
F10/R10	19613	3458	54.14	0.70	148	0.89
**Extraction Procedure**						
Control	27244	8213	0	1.30	415	0.96
Frozen	30152	9878	0	1.32	407	0.93
Frozen/bead beaten	24419	7466	0	1.35	387	0.92

The table shows the total number of reads, the number remaining after filtering and the % of non-specific reads (those not aligning to 16S) and chimeras (see methods for chimera detection) present when different annealing temperatures, primer pairs and extraction procedures were employed. The figures show the combined results of two replications.

a Average of the two replications.

### Effect of primer length

We designed various primers patterned on conserved regions of the 16S rRNA gene, flanking variable regions V1–V3: a pair of primers of conventional length, F20 and R19, three short forward primers, F10, F12 and F14, and one short reverse primer R10 (Table 2) The shorter primers were employed to try and capture sequences that differed in the 3′ region of universal binding sites. In the case of the short reverse primer (R10), the inosines, present in the full length primer, were replaced with conventional bases. The reason for this was that a primer containing only 6 non redundant bases was considered to be too promiscuous. Although the primer would potentially amplify fewer sequences, it would still fulfill its purpose and identify target sequences that differed in the region complementary to the 3′ end of the longer primer sequence. Various combinations of primers were evaluated under relatively permissive PCR conditions (annealing temperature 40°C). PCR products were obtained when the F20/R19, F14/R19 and F10/R10 combinations were used. However, the F10/R19 and F12/R19 combinations failed to produce a PCR product, even when the annealing temperature was lowered to 30°C.

**Table 2:** The sequence of the region of the primers complementary to the 16S rRNA gene used in this study.

Primer	reference	sequence
F20(27F-YM)	[6]	AGAGTTTGATYMTGGCTCAG
F14	[11]	AGAGTTTGATYMTG
F12	[11]	AGAGTTTGATYM
F10	[11]	AGAGTTTGA
*Bifidobacterium* 1		AG**G**GTT**C**GATTCTGGCTCAG
R19(I-533R)	[17]	TIACCGIIICTICTGGCAC
R10	This study	TTACCGCGGC

The full length forward primer corresponds to bases 8–27 and the reverse primer to bases 515–534 (using the *E. coli* numbering system).

1 The sequence of the corresponding region of the *Bifidobacterium* 16S rRNA gene is also shown for comparison, with bases in bold showing mis-matches with the primer sequences.

In most amplicon libraries we saw little non-specific amplification of sequences that did not originate from 16S rRNA genes, despite the relatively low annealing temperature used in these experiments (Table 1). The one notable exception was obtained with the F10/R10 primer combination–here, over 50% of reads represented non-specific sequences. We detected no significant differences in the number of chimeras in the amplicon libraries obtained from any of the primer pairs (Table 1).

**Figure 1:**
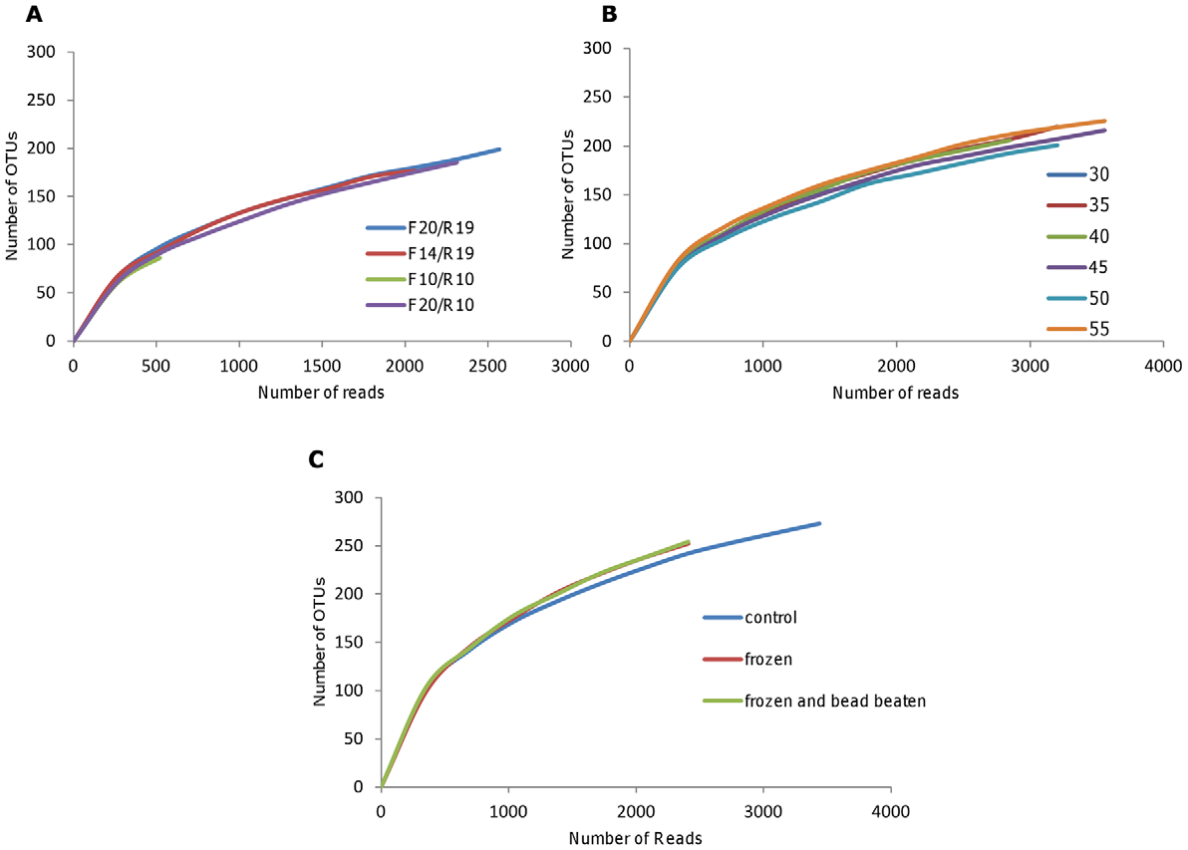
Rarefaction curves of the 97% OTUs for the different experimental protocols. At each sampling depth, the average number of OTUs is shown (n=2) (a) Different primer pairs (b) Different annealing temperatures (c) Different extraction procedures.

**Figure 2:**
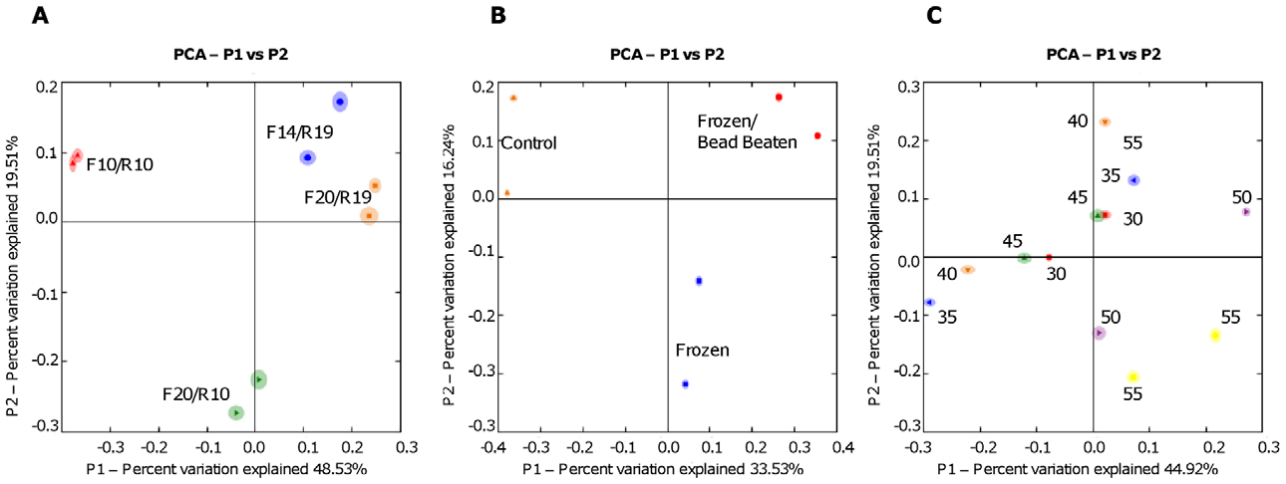
Hierarchical clustering visualizing the similarity of the bacterial communities as judged by pyrosequencing using different experimental procedures. All bootstrap values greater than 90% are displayed on branch lines. (a) Different primer pairs (b) Different annealing temperatures (c) Different extraction procedures.

**Figure 3:**
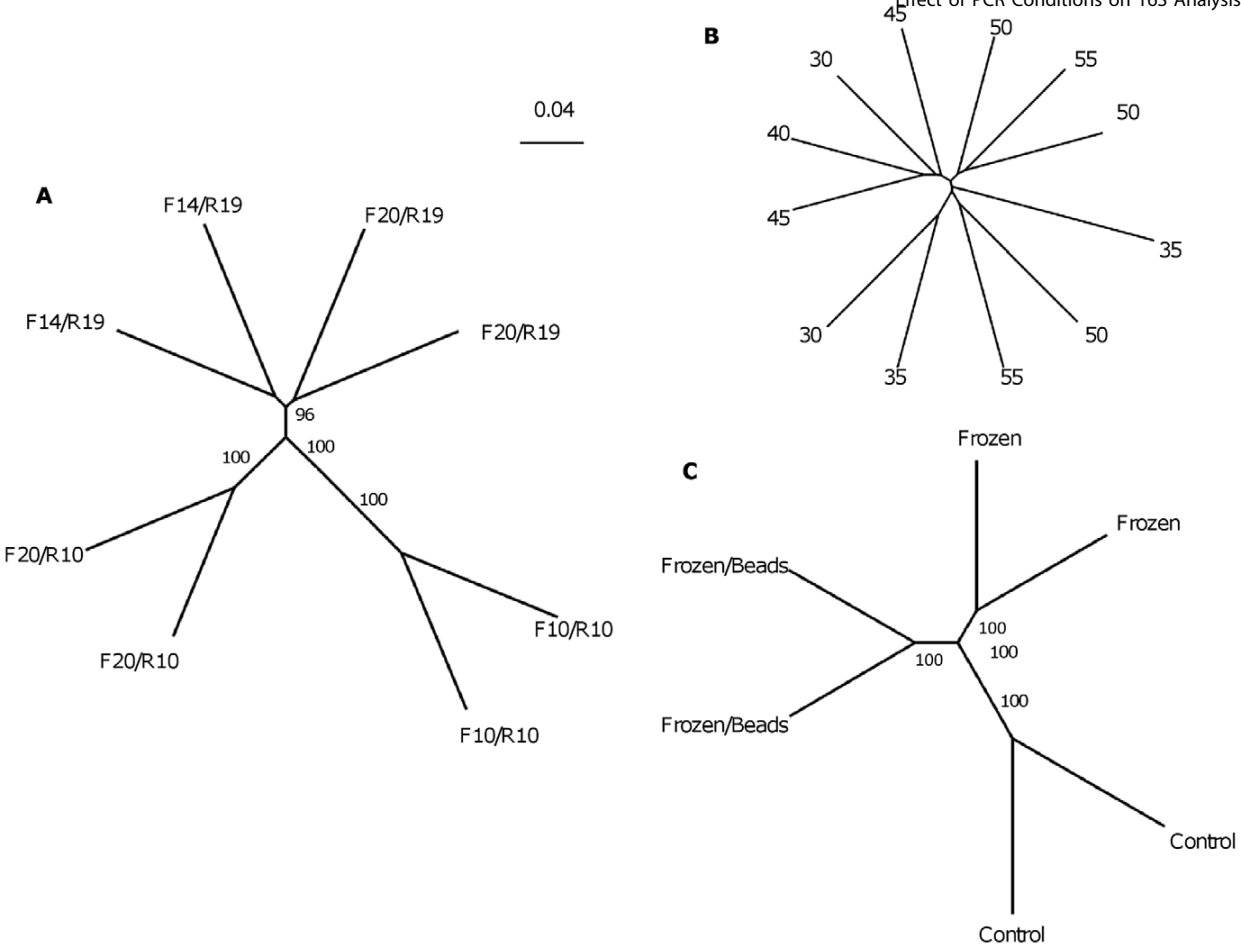
PCA visualization of the differences between the observed bacterial communities as judged by pyrosequencing generated by different experimental procedures. (a) Different primer pairs (b) Different annealing temperatures (c) Different extraction procedures.

Use of short primers had little effect on species richness (Figure 1a) or species evenness as measured by Simpsons' Index (Table 1), nor did their use lead to the identification of any significant novel OTUs. However, there were substantial differences in relative abundance of OTUs (Table 3) and the community structures of the libraries created by the different primer combinations were clearly different as judged by UGPMA clustering (Figure 2a) and PCA analysis (Figure 3a). In particular, *Bifidobacterium* could not be detected at all by the shorter forward primers, but could be detected at higher abundance using the shorter reverse primer. In addition OTUs corresponding to *Lactobacillus* were more abundant when the shorter reverse primer was used.

**Table 3:** OTUs which differed significantly when different primer pairs were employed in the PCR.

		Primers used^b^			
Consensus lineage	P Value^a^	F14/R19	F20/R10	F20/R10	F10/R10
Bacteroidetes; Bacteroidia; Bacteroidales; Rikenellaceae; Alistipes;	0.035	20.84	14.20	25.14	5.89
Proteobacteria; Epsilonproteobacteria; Campylobacterales; Campylobacteraceae; Campylobacter;	0.001	3.75	4.15	4.27	11.91
Actinobacteria; Actinobacteria; Actinobacteridae; Bifidobacteriales; Bifidobacteriaceae;	0.003	0.00	5.98	3.31	0.00
Firmicutes; Bacilli; Lactobacillales; Lactobacillaceae; Lactobacillus;	0.004	2.76	5.12	2.61	3.48
Firmicutes; Bacilli; Lactobacillales; Lactobacillaceae; Lactobacillus;	0.031	1.45	3.96	2.35	1.63
Firmicutes; Clostridia; Clostridiales; Ruminococcaceae; Faecalibacterium;	0.001	3.63	0.64	2.17	1.42
Firmicutes; Clostridia; Clostridiales; Ruminococcaceae;	0.012	1.38	3.08	1.78	3.54
Firmicutes; Clostridia; Clostridiales;	0.005	1.56	1.61	0.85	4.25
Firmicutes; Clostridia; Clostridiales; Veillonellaceae; Allisonella;	0.005	0.79	1.07	0.80	5.03
Firmicutes; Clostridia; Clostridiales; Lachnospiraceae;	0.041	1.05	1.22	0.74	0.37
Firmicutes; Clostridia; Clostridiales; Ruminococcaceae; Oscillibacter;	0.030	0.77	1.01	0.65	1.38
Firmicutes; Clostridia; Clostridiales; Lachnospiraceae;	0.002	0.41	0.07	0.51	0.06
Firmicutes; Clostridia; Clostridiales; Ruminococcaceae; Faecalibacterium;	0.015	1.28	0.20	0.47	0.25
Firmicutes; Bacilli; Lactobacillales; Lactobacillaceae; Lactobacillus;	0.016	0.84	1.05	0.44	1.12
Firmicutes; Clostridia; Clostridiales; Ruminococcaceae; Faecalibacterium;	0.047	0.39	0.08	0.23	0.00
Proteobacteria;	0.008	0.08	0.37	0.19	0.00
Bacteroidetes; Bacteroidia; Bacteroidales; Porphyromonadaceae; Butyricimonas;	0.004	0.25	0.12	0.18	0.00
Bacteroidetes; Bacteroidia; Bacteroidales; Rikenellaceae; Alistipes;	0.037	0.16	0.05	0.15	0.00
Bacteroidetes; Bacteroidia; Bacteroidales; Rikenellaceae; Alistipes;	0.024	0.19	0.09	0.13	0.00
Firmicutes; Erysipelotrichi; Erysipelotrichales; Erysipelotrichaceae; Holdemania;	0.004	0.02	0.11	0.13	0.00
Firmicutes; Clostridia; Clostridiales; Ruminococcaceae; Faecalibacterium;	0.005	0.37	0.02	0.13	0.06
Actinobacteria; Actinobacteria; Coriobacteridae; Coriobacteriales; Coriobacterineae;	0.001	0.00	0.14	0.10	0.00
Firmicutes; Clostridia; Clostridiales; Ruminococcaceae; Lactonifactor;	0.001	0.08	0.04	0.10	0.00
Firmicutes;	0.006	0.00	0.39	0.08	0.25
Firmicutes; Clostridia; Clostridiales;	0.000	0.04	0.00	0.07	0.00
Firmicutes; Clostridia; Clostridiales; Ruminococcaceae; Anaerotruncus;	0.011	0.04	0.04	0.05	0.13
Firmicutes; Clostridia;	0.002	0.00	0.00	0.04	0.00
Firmicutes; Clostridia; Clostridiales; Ruminococcaceae;	0.039	0.06	0.16	0.04	0.00
Firmicutes; Clostridia; Clostridiales; Ruminococcaceae;	0.000	0.00	0.00	0.03	0.00
Firmicutes;	0.000	0.00	0.00	0.03	0.00
Unclassified	0.000	0.00	0.00	0.03	0.00
Firmicutes; Clostridia; Clostridiales;	0.000	0.00	0.00	0.03	0.00
Firmicutes; Clostridia; Clostridiales;	0.023	0.15	0.14	0.03	0.31
Firmicutes; Clostridia; Clostridiales;	0.021	0.00	0.04	0.03	0.00
Firmicutes; Clostridia; Clostridiales; IncertaeSedisXIII;	0.021	0.00	0.04	0.03	0.00
Actinobacteria; Actinobacteria; Coriobacteridae; Coriobacteriales; Coriobacterineae;	0.044	0.00	0.00	0.03	0.00
Firmicutes; Clostridia; Clostridiales; Ruminococcaceae; Lactonifactor;	0.044	0.00	0.00	0.03	0.00
Unclassified	0.044	0.00	0.00	0.03	0.00
Firmicutes; Clostridia; Clostridiales; Ruminococcaceae; Anaerotruncus;	0.044	0.00	0.00	0.03	0.00
Firmicutes; Clostridia; Clostridiales; Lachnospiraceae;	0.044	0.00	0.00	0.03	0.00
Firmicutes; Clostridia; Clostridiales; Ruminococcaceae; Subdoligranulum;	0.009	0.00	0.02	0.02	0.13
Firmicutes; Clostridia; Clostridiales; Lachnospiraceae;	0.003	0.17	0.02	0.02	0.00
Firmicutes; Clostridia; Clostridiales;	0.006	0.00	0.11	0.02	0.00
Firmicutes; Clostridia; Clostridiales; Ruminococcaceae;	0.009	0.10	0.05	0.02	0.00
Firmicutes; Clostridia; Clostridiales; Ruminococcaceae;	0.046	0.02	0.09	0.02	0.00
Firmicutes; Clostridia; Clostridiales; Ruminococcaceae; Anaerofilum;	0.001	0.08	0.00	0.01	0.00
Unclassified	0.002	0.10	0.04	0.01	0.00
Unclassified	0.006	0.04	0.07	0.01	0.00
Firmicutes; Clostridia; Clostridiales;	0.013	0.02	0.02	0.01	0.13
Firmicutes; Clostridia; Clostridiales; Lachnospiraceae;	0.017	0.00	0.04	0.01	0.00
Firmicutes; Clostridia; Clostridiales;	0.017	0.00	0.04	0.01	0.00
Firmicutes; Clostridia; Clostridiales;	0.017	0.00	0.04	0.01	0.00
Firmicutes; Clostridia; Clostridiales; Lachnospiraceae;	0.000	0.00	0.00	0.00	0.13
Firmicutes; Clostridia; Clostridiales; Ruminococcaceae; Oscillibacter;	0.000	0.00	0.05	0.00	0.13
Firmicutes; Clostridia; Clostridiales; Ruminococcaceae; Subdoligranulum;	0.000	0.04	0.00	0.00	0.00
Unclassified	0.001	0.00	0.04	0.00	0.00
Firmicutes; Clostridia; Clostridiales; Ruminococcaceae;	0.005	0.00	0.05	0.00	0.00

a The P value was calculated in QIIME (see methods).

b values show the average percentage of reads for each primer pair (n=2) in the OTU compared to the total number of filtered reads in the sample.

### Effect of annealing temperature

We explored the effects of annealing temperature on a PCR that included standard-length primer pair, F20/R19. The annealing temperature was reduced in 5°C decrements from 55° to 30°C. Surprisingly, even at an annealing temperature of 30°C, a distinct PCR product was obtained and could be visualized with little smearing on agarose gel electrophoresis. As with different length primers, there was little difference in species richness or evenness at different annealing temperatures (Table 1 and Figure 1b). Indeed temperature had little correlation to library composition as judged by UGPMA clustering and PCA analysis (Figure 2b and 3b). However, a small number of OTUs (13 out of 529) showed a significant correlation with annealing temperature (Table 4). In particular those from the genera *Bifidobacterium*, *Faecalibacterium* and *Campylobacter*, increased in relative abundance as the annealing temperature decreased.

**Table 4:** OTUs which showed correlation with annealing temperature.

			Annealing Temperature (°C)^c^					
Consensus Lineage	P value^a^	R^b^	30	35	40	45	50	55
Proteobacteria;	0.0001	0.90	0.09	0.20	0.19	0.19	0.27	0.29
Actinobacteria; Actinobacteria; Actinobacteridae; Bifidobacteriales; Bifidobacteriaceae;	0.0006	−0.84	3.40	3.97	3.31	2.77	1.30	0.63
Unclassified	0.0017	0.80	0.26	0.45	0.44	0.44	0.59	0.73
Proteobacteria; Epsilonproteobacteria; Campylobacterales; Campylobacteraceae; Campylobacter;	0.0020	−0.80	4.33	4.87	4.27	3.97	2.03	2.37
Firmicutes; Clostridia; Clostridiales; Veillonellaceae; Allisonella;	0.0151	0.68	0.92	0.85	0.80	0.89	1.13	1.16
Firmicutes; Clostridia; Clostridiales; Ruminococcaceae; Oscillibacter;	0.0152	0.68	0.03	0.02	0.03	0.07	0.07	0.09
Firmicutes; Clostridia; Clostridiales;	0.0156	−0.68	0.31	0.40	0.41	0.20	0.14	0.19
Firmicutes; Clostridia; Clostridiales; Ruminococcaceae; Faecalibacterium;	0.0187	−0.66	2.40	2.36	2.17	1.76	2.32	1.21
Firmicutes; Clostridia; Clostridiales;	0.0190	−0.66	0.22	0.26	0.12	0.13	0.13	0.10
Firmicutes; Clostridia; Clostridiales; Ruminococcaceae;	0.0264	0.64	0.06	0.09	0.11	0.22	0.16	0.26
Firmicutes; Clostridia; Clostridiales; Lachnospiraceae;	0.0343	−0.61	1.13	0.96	1.01	1.10	0.79	0.67
Firmicutes; Clostridia; Clostridiales; Lachnospiraceae;	0.0368	0.61	0.31	0.25	0.33	0.37	0.40	0.44
Proteobacteria; Epsilonproteobacteria; Campylobacterales; Helicobacteraceae; Helicobacter;	0.0482	−0.58	3.31	3.76	2.86	2.74	2.60	2.65

a The P value was calculated in QIIME (see methods).

b Pearson's r value with −1 or +1 indicating a perfect negative or positive correlation respectively and 0 indicating no correlation.

c the average percentage of reads for each annealing temperature (n=2) in the OTU compared to the total number of filtered reads in the samples.

### Effect of extraction procedure

We evaluated results obtained from three different extraction procedures on pairs of caecal contents that had been flash-frozen after harvesting and stored at −70°C. These samples were defrosted, homogenized and then dispensed into 0.22 ml aliquots. For each pair, one control aliquot was subjected to chemical lysis on thawing, a second aliquot was subjected to a second free-thaw step before chemical lysis, while a third was freeze-thawed and then subjected to mechanical lysis (bead-beating) before chemical lysis.

All three approaches recovered the same OTUs and species evenness and richness were similar (Table 1 and Figure 1c). However, the apparent community structures produced by the different extraction procedures differed considerably, with even the refreezing step producing a distinct library of sequences (Figures 2c and 3c). In addition, we found significant differences in the relative abundance of OTUs. All those that increased in relative abundance in the re-frozen and bead-beaten samples belonged to the *Firmicutes*, with the exception of one unclassified OTU (Table 5). One OTU from the genus *Faecalibacterium* showed a four-fold increase in the bead-beaten samples, although the relative abundance of other OTUs from this genus was not affected by bead beating or re-freezing (Table S4). Two OTUs corresponding to *Bacteroides* decreased in abundance in the bead-beaten samples, but, perhaps surprisingly, there was no increase in prevalence of *Proteobacteria* such as *E. coli* and *Campylobacter* in the control (chemical lysis) samples.

**Table 5:** OTUs which showed significant differences when different extraction methods were employed.

		Extraction Procedure^b^		
Consensus lineage	P Value^a^	Control	Frozen	Bead
Firmicutes; Clostridia; Clostridiales; Veillonellaceae; Megamonas;	0.018	15.87	23.89	25.90
Firmicutes; Clostridia; Clostridiales; Ruminococcaceae; Faecalibacterium;	0.032	2.00	3.59	7.28
Firmicutes; Clostridia; Clostridiales; Lachnospiraceae;	0.029	2.71	4.96	3.40
Firmicutes; Bacilli; Lactobacillales; Lactobacillaceae; Lactobacillus;	0.003	0.74	0.56	2.02
Firmicutes; Clostridia; Clostridiales; Ruminococcaceae;	0.003	0.52	0.92	1.95
Firmicutes; Clostridia;	0.035	2.57	1.87	1.26
Firmicutes; Clostridia; Clostridiales; Lachnospiraceae;	0.020	2.36	1.71	1.01
Bacteroidetes; Bacteroidia; Bacteroidales;	0.029	1.74	1.10	0.89
Firmicutes; Clostridia; Clostridiales;	0.013	1.02	1.75	0.54
Firmicutes; Clostridia; Clostridiales; Ruminococcaceae;	0.015	0.37	0.94	0.47
Firmicutes; Clostridia; Clostridiales;	0.039	0.71	0.52	0.41
Unclassified	0.034	0.69	0.55	0.38
Firmicutes; Clostridia; Clostridiales; Lachnospiraceae; Roseburia;	0.026	0.02	0.04	0.26
Bacteroidetes; Bacteroidia; Bacteroidales; Bacteroidaceae; Bacteroides;	0.036	0.60	0.36	0.25
Firmicutes; Clostridia; Clostridiales; Lachnospiraceae;	0.004	0.00	0.00	0.25
Firmicutes; Clostridia; Clostridiales;	0.042	0.41	0.23	0.19
Firmicutes; Clostridia;	0.019	0.03	0.00	0.18
Firmicutes; Clostridia; Clostridiales; Ruminococcaceae; Oscillibacter;	0.018	0.14	0.06	0.12
Firmicutes; Clostridia; Clostridiales; Ruminococcaceae;	0.042	0.08	0.15	0.11
Firmicutes; Clostridia; Clostridiales;	0.001	0.22	0.05	0.09
Firmicutes; Clostridia; Clostridiales; Lachnospiraceae; Syntrophococcus;	0.002	0.00	0.01	0.09
Firmicutes; Clostridia; Clostridiales;	0.004	0.00	0.00	0.08
Firmicutes; Clostridia; Clostridiales; Lachnospiraceae; Parasporobacterium;	0.001	0.00	0.00	0.07
Firmicutes; Bacilli; Lactobacillales; Streptococcaceae; Streptococcus;	0.011	0.04	0.02	0.07
Firmicutes; Clostridia;	0.022	0.00	0.05	0.07
Firmicutes; Clostridia; Clostridiales; Ruminococcaceae;	0.024	0.04	0.01	0.07
Firmicutes; Clostridia; Clostridiales; Ruminococcaceae;	0.009	0.07	0.19	0.06
Firmicutes; Clostridia; Clostridiales; Ruminococcaceae; Acetanaerobacterium;	0.045	0.06	0.01	0.04
Firmicutes; Bacilli; Lactobacillales; Lactobacillaceae; Lactobacillus;	0.004	0.00	0.00	0.03
Unclassified	0.004	0.00	0.00	0.03
Firmicutes; Clostridia; Clostridiales;	0.004	0.00	0.00	0.03
Firmicutes; Clostridia; Clostridiales; Lachnospiraceae;	0.031	0.11	0.05	0.01
Unclassified	0.040	0.07	0.07	0.01
Firmicutes; Clostridia; Clostridiales;	0.001	0.00	0.02	0.00
Firmicutes; Clostridia; Clostridiales; Lachnospiraceae;	0.001	0.00	0.02	0.00
Firmicutes; Clostridia; Clostridiales;	0.001	0.00	0.02	0.00
Firmicutes; Clostridia; Clostridiales; Ruminococcaceae; Anaerotruncus;	0.008	0.04	0.00	0.00
Firmicutes; Clostridia; Clostridiales; Ruminococcaceae; Oscillibacter;	0.008	0.04	0.00	0.00
Firmicutes; Clostridia; Clostridiales;	0.008	0.04	0.00	0.00
Firmicutes;	0.008	0.04	0.00	0.00
Firmicutes;	0.008	0.04	0.00	0.00
Firmicutes; Clostridia; Clostridiales; Ruminococcaceae; Acetanaerobacterium;	0.009	0.03	0.00	0.00
Firmicutes; Clostridia; Clostridiales; Eubacteriaceae; Eubacterium;	0.009	0.03	0.00	0.00
Firmicutes; Clostridia; Clostridiales;	0.013	0.03	0.02	0.00
Unclassified	0.018	0.04	0.09	0.00
Unclassified	0.028	0.03	0.15	0.00
Firmicutes; Clostridia; Clostridiales; Ruminococcaceae; Oscillibacter;	0.038	0.25	0.22	0.00

a The P value was calculated in QIIME (see methods).

b the average percentage of reads for each primer pair (n=2) in the OTU compared to the total number of filtered reads in the samples.

## Discussion

Isenbarger and colleagues reported that use of short 16S rRNA gene “miniprimers” led to greater observed diversity in bacterial populations from soil and microbial mats [9]. However, in our hands, when twinned with a high-throughput sequencing approach, this ‘miniprimer’ strategy provided little advantage over use of conventional primers in terms of species richness in the chicken caecal microbiome, particularly as more than half the amplicons obtained with the F10/R10 miniprimer pair were non-specific.

It is not clear why our findings differ from those in the earlier study. One potentially important difference is that, although the template-specific regions in our mini-primers were short, the primers incorporated adapter and bar-coding sequences at their 5′ ends. One explanation for differences in OTU abundance in reactions where full-length and mini-primers are used might be that these additional sequences provide complementarity in the early rounds of amplification to selected 16S rRNA gene sequences. To investigate this possibility, we retrieved full-length 16S rRNA genes sequences from the public databases for two Lactobacillus OTUs that increased in relative abundance when the R10 primer was used. However, on scrutinizing these sequences, we found no matches to non-template-specific sequences in the R10 primer. Another possibility is that the barcoding and adapter extensions altered the dynamics of the PCR after the initial rounds of amplification.

We were also surprised to find that annealing temperature had little effect on the number of reported OTUs or on the number of nonspecific and chimeric products, even when the temperature was reduced to 30°C. This contrasts with previous studies where lowering the annealing temperature led to apparent increases in the diversity of sequences obtained from a termite gut [16], a cattail rhizoplane sample [8] and a compost sample [15]. One plausible explanation for this discrepancy is that our primers incorporated degenerate bases and inosine at selected, less conserved positions, thereby decreasing the likelihood of mismatches. Hence it is possible that there were very few targets that contained mismatches in the primer target region. This theory is supported by the fact that no new sequences were obtained with the short primers, implying there were no abundant target sequences which differed in the 5′ 10 base pairs of either primer. However, the fact that few non-16S rRNA gene sequences and chimeras were generated at the lowest annealing temperature (30°C) implies that this technique may be worth pursuing in analysing the microbial content of environmental samples other than the chicken caecum, where 16S rRNA gene sequences may be more divergent.

Nonetheless, there were some significant differences in the relative abundance of different OTUs at different annealing temperatures. For example, the abundance of OTUs from the genus *Bifidobacterium* increased at lower annealing temperatures. This can be explained by the existence of two mismatches in the *Bifidobacterium* 16S rRNA gene that correspond to the 5′ end of the primer we used (Figure 1). This is consistent with previous studies on mixed templates, one of which perfectly matches the primer, while the other contains mismatches [7], [8], [15]. In such studies, at the higher annealing temperature, the perfectly matching template is preferentially amplified, but this bias disappears as the temperature is lowered But not all changes in abundance at lower annealing temperatures can be explained this way–for example, we found no evidence of such mismatches in e.g. *Faecalibacterium* and *Campylobacter* 16S rRNA gene sequences. Therefore as with the short primers, the preferential amplification at different temperatures is likely to be due to sequence differences outside the immediate primer target region. In a previous study [6] linear amplification using only the 27f-YM (F20) primer preferentially amplified the perfectly matching *Lactobacillus* 16S rRNA gene over *Gardnerella*, a member of the *Bifidobacteriales*, which contained two mismatches at the 5′ end (Table 2). A bias was still observed when the annealing tempereature was lowered to 48°C and it was suggested that lowering the annealing temperature may not be sufficient to overcome the mismatches in the primer sequence. However, in this study, lowering the annealing temperature below 48°C further increased the percentage of *Bifidobacterium* 16S rRNA gene sequences obtained (Table 4), suggesting that amplification bias can be further reduced by decreasing the annealing temperature below 48°C.

In line with a previous survey [2], we found that mechanical lysis of bacterial cells by bead beating led to an increased relative abundance of Gram-positive taxa in our samples. However, this effect did not appear to be uniform even within members of the same genus; for example, not all OTUs from Faecalibacterium and Lactobacillus followed the same trend (Table S4). Thus the effect of mechanical disruption is likely to be species specific and hence it would be difficult to predict specific species that would be under-represented when more gentle chemical lysis was employed. However, representation of some gram positive (Firmicutes) OTUs decreased in the bead-beaten preparations as well as two Bacteroides OTUs (Table 5). These probably represent species that can be more easily lysed under the chemical lysis procedure and hence are over represented in those samples. In addition, such cells may lyse early on during the bead beating step resulting in a longer period in which the DNA can be sheared or degraded. It is therefore surprising that a number of Firmicutes and no Proteobacteria were among this group.

In conclusion, we found that variations in primer length, annealing temperature and extraction protocol had only minor effects on species richness in our samples and revealed no new significant OTUs additional to those found under standard conditions. Effects were seen on the relative abundance of some OTUs, but it remains unclear which protocol yielded the most accurate quantitative description of this microbial community, given the absence of any gold standard for enumerating sequences by taxa for such complex communities.

## Materials and Methods

### Sample Extraction and PCR

Two chicken caecal samples were collected from two 42 day old Ross broilers, that had been housed indoors under standard commercial conditions. Birds were euthanized by cervical dislocation, the caeca removed and transported to the laboratory on ice. The caecal surface was disinfected with 70% ethanol, a longitudinal incision made with a scalpel and the edges pulled back. Contents were removed into a sterile 15 ml Corning tube, flash-frozen in liquid nitrogen and stored at −70°C.

At the outset of the primer length/annealing temperature experiments, a section of a single frozen caecal content (200 mg) was taken and DNA extracted using the QIAampDNA Stool Mini Kit (Qiagen, Crawley, UK), following manufacturer's instructions. This sample of DNA was then used for all PCRs in the primer length/annealing temperature experiments. To study extraction procedures, a separate frozen caecal sample was taken, mixed with an equal volume of buffer (100 mM EDTA; 25 mM TRIS-HCl pH 8.0; 50 mM glucose) and homogenized by pipetting up and down using a 5 ml pipette. The homogenized caecal sample was then dispensed into six 220 µl aliquots. Two of these aliquots were used immediately for DNA extraction whilst four were flash-frozen again in a dry ice/ethanol bath before extraction. DNA extraction was again performed using the QiAMP DNA Stool Mini Kit, but two of the aliquots were subjected to an additional bead-beating step, which was included after the addition of buffer AL to the sample. This step involved adding 0.2 g of 100–300 µM acid washed glass beads (Sigma, Poole, UK) followed by disruption with 2×30 sec pulses at speed setting of 6.2 m/s in a FastPrep FP120 (Qbiogene, Cambridge, UK). DNA was measured using a nanodrop 1000 (ThermoScientific, UK)

PCR was performed with 200 ng DNA in a 25 µl reaction and 0.8 µM of each primer using 12.5 µl of 2× Extensor Master Mix 1 (Abgene, Espom, UK). Cycling conditions (using a Thermo Hybaid MBS 0.2G cycler) were: 94°C for 3 min, 30 cycles of 94°C for 30 sec; 30–55°C (see below) for 30 sec; 68°C for 1 min with a final extension step of 68°C for 5 min. For the extraction experiment, an annealing temperature of 55°C was used and for the short primers this was lowered to 40°C. Annealing temperatures between 30°C and 55°C in 5°C increments were used to ascertain the effect of annealing temperature on OTUs produced. The full length forward primer (F20) was based on 27F-YM and contains degenerate bases at positions 11 (C or T) and 12 (C orA), which accommodate differences in the 16S rRNA gene of the *Campylobacterales*, *Sphingomonadales* and *Actinobacteria* as well as many enteric bacteria [6]. The full length revesrse primer was based on I533-R which contains four inosine residues (that can pair with any base) and permits amplification of *Verrucomicrobia* and candidate division OP11 16S rRNA genes [17]. A range of primers that differed in the length of the region complementary to the 16S rRNA gene target were used in miniprimer experiments (Table2). In addition, the forward primers had a 10-bp barcode followed by the adapter sequence (CGTATCGCCTCCCTCGCGCCATCAG) at the 5′ end. Reverse primers contained the adapter sequence (CTATGCGCCTTGCCAGCCCGCTCAG) at the 5′ end. For each annealing temperature and primer combination PCRs were carried out in duplicate. Each extraction procedure was carried out in duplicate and a single PCR was performed on each replicate. Table S1 shows a summary of the conditions used in each experiment and sequences of the barcodes and primers used. The PCR Amplicons were purified using AmpPure beads (Beckman Coulter, Takeley, UK) and quantified by fluorimetry using PicoGreen Quant IT (Invitrogen, Paisley, UK) following manufacturer's instructions.

### 454 Sequencing Protocols

Amplicons were diluted and pooled so that the concentration of each amplicon in the pool was 10^7^ molecules µl^−1^. Two pools were used on two sectors of a 454 chip (Table S1). The prepared pooled samples were primed for sequencing as per 454 emPCR manufacturer's protocols. The copy to bead ratios were adjusted for each pool to ensure between 1,500,000 and 6,000,000 enriched beads were collected for sequencing. Sequencing was carried out on the Roche FLX Titanium instrument using the protocol recommended by Roche 454 for a 4-region picotiter plate. The resulting SFF files were used for downstream analysis

### Bioinformatic Procedures

Sequences were filtered based on the method used in Amplicon Noise [18]. Sequences were truncated where flow signals were less than 0.7 and all sequences were trimmed to 400 flows (around 250 bp in length). The sequences were then processed with Amplicon Noise using the PyroNoiseM program with a cut off of 0.01 and a precision of 60 and SeqNoiseM with a cut off of 0.08 and a precision of 25. After de-noising the flow data (the PyroNoiseM step), the barcode and primer sequence were removed and sequences were truncated to 220 bp before the SeqDistM step. Chimeras were then removed using Perseus [16] using the default settings of an alpha value of −6.6925 and a beta value of 0.5625.

Sequences were then clustered using Esprit-Tree [19] at a distance threshold of 0.03 to form OTUs. Instead of picking a single read to represent the OTU, which may not be representative of all the sequences assigned to that OTU, a consensus sequence of the constituent reads was constructed. This was achieved by aligning all reads in an OTU using Muscle [20] The majority base at each position of the alignment was then used to form the consensus sequence. This consensus sequence was used to assign the OTU to a taxonomic lineage using the RDP database [21] with a bootstrap cut-off of 50% as recommended for sequences less than 250 bp in length. In addition all OTUs were searched by BLAST against the SILVA database (SSUREf 104) with e value cut-off of 1 e-5. OTUs below this cut off were considered non 16S rRNA genes and thus removed from subsequent analysis.

The resultant OTUs, frequencies and taxonomic grouping were formatted into an OTU table that was compatible with the QIIME pipeline [22]. QIIME was used to calculate Simpsons' index and construct rarefaction curves. Dendrograms depicting the similarity of bacterial communities were constructed by using the jackknifed_beta_diversity script. The script used the Bray Curtis method to compute a similarity matrix and then un-weighted pair group method with arithmetic mean (UGPMA) to cluster the results. Jacknifed support was included to account for the different sampling depths. A hundred rarefied tables were generated at sample size that corresponded to the number of reads in the smallest sample. Distance matrixes were computed for each rarefied table and compared to the full tree in order to produce bootstrap values. PCA analysis was also performed on the rarefied tables using QIIME. OTUs which differed significantly between treatments were identified by using the QIIME otu_category_significance script, with –s correlation option for annealing temperature and –s ANOVA option for primer length and extraction procedures.

## Supporting Information

Table S1 Summary of the experimental conditions used to produce and sequence the amplicons in each PCR reaction.
(XLSX)

Table S2 The number of reads in each OTU obtained by the different PCR primer pairs and their assignment using the RDP classifier and the closest BLAST match from the SILVA and NCBI nr databases.
(XLSX)

Table S3 The number of reads in each OTU obtained by different annealing temperatures in the PCR and their assignment using the RDP classifier and the closest BLAST match from the SILVA and NCBI nr databases.
(XLSX)

Table S4 The number of reads in each OTU obtained from each extraction procedure and their assignment using the RDP classifier and the closest BLAST match from the SILVA and NCBI nr databases.
(XLSX)
